# EGR3 as a dual tumor-immune regulator: a machine learning-driven prognostic target for cold breast cancer

**DOI:** 10.3389/fimmu.2025.1627133

**Published:** 2025-12-15

**Authors:** Qianxue Wu, Daqiang Song, Jian Yue, Benhua Li, Junge Gong, Xiang Zhang

**Affiliations:** 1 Department of Breast and Thyroid Surgery, The First Affiliated Hospital of Chongqing Medical University, Chongqing, China; 2 Department of Breast Surgery, Gaozhou People’s Hospital, Gaozhou, Guangdong, China; 3 Department of Clinical Laboratory, The Second People’s Hospital of Liangshan yi Autonomous Prefecture, Xichang, Sichuan, China; 4 Clinical Molecular Medicine Testing Center, The First Affiliated Hospital of Chongqing Medical University, Chongqing, China

**Keywords:** breast cancer, multi-omics integration, tumor-immune, machine learning, EGR3

## Abstract

**Background:**

Breast cancer heterogeneity necessitates robust prognostic biomarkers and therapeutic targets. This study aimed to identify key molecular drivers through integrative multi-omics approaches and validate their clinical relevance.

**Methods:**

We combined differential expression analysis, weighted gene co-expression network analysis (WGCNA), and machine learning (StepCox-Random Survival Forest [RSF]) to screen prognostic signatures across TCGA, GEO (GSE42568, GSE9893, GSE7390), and METABRIC datasets. Immune microenvironment characterization utilized ESTIMATE, CIBERSORT, and functional enrichment analyses. Mechanistic validation included single-cell RNA sequencing, *in vitro/in vivo* experiments, and clinical cohort profiling.

**Results:**

WGCNA identified 102 hub genes linked to breast cancer progression. Machine learning optimization yielded a 3-gene signature (EGR3, RECQL4, MMP1) with superior prognostic stratification. Multi-cohort validation confirmed signature robustness. The C2 subtype, defined by high-risk scores, exhibited an immunosuppressive microenvironment with elevated PD-L1/LAG3/TIGIT and M2 macrophage enrichment. EGR3 emerged as a pivotal tumor suppressor: its expression inversely correlated with tumor stage and positively associated with CD8^+^ T cell infiltration. EGR3-high patients showed prolonged survival and enhanced immunotherapy response. Functional studies demonstrated EGR3 overexpression suppressed tumor growth and activated CD8^+^ T cells.

**Conclusion:**

Our integrative framework established a machine learning-optimized 3-gene prognostic model with cross-platform reliability. EGR3 was validated as a dual-function regulator of tumor suppression and immunomodulation, offering a novel therapeutic target for breast cancer, particularly in immunologically “cold” triple-negative subtypes.

## 1 Introduction

Breast cancer remains a leading cause of cancer-related mortality among women worldwide, with its marked heterogeneity and complex molecular mechanisms posing significant challenges for accurate prognostic prediction and therapeutic strategy development (1). While advances in genomic and transcriptomic biomarker research have improved risk stratification, current prognostic models face limitations in clinical translation, including insufficient generalizability across datasets, inadequate resolution of dynamic tumor microenvironment interactions, and a paucity of mechanistic insights into key driver genes (2). The integration of multi-omics analyses and artificial intelligence has emerged as a transformative approach to identify robust prognostic signatures and elucidate their biological underpinnings, offering potential to refine precision oncology in breast cancer (3).

Despite growing applications of weighted gene co-expression network analysis (WGCNA) and machine learning algorithms in cancer biomarker discovery (4), critical gaps persist in effectively leveraging multi-center cohort data to optimize model performance and decipher the immune-regulatory mechanisms of prognostic gene signatures. Previous studies predominantly focused on single-omics approaches or conventional statistical methods for model construction, often overlooking tumor microenvironment heterogeneity and lacking experimental validation of candidate genes (5, 6). For instance, while Cai et al. applied WGCNA to identify HER2-associated modules, they omitted clinical validation of prognostic utility (7). Similarly, Zhang et al. employed random survival forest (RSF) without prior co-expression network filtering, resulting in gene lists lacking mechanistic coherence (8). Moreover, the functional roles of immunomodulatory molecules—particularly early growth response factors like EGR3—in breast cancer progression remain poorly defined, further restricting clinical translation.

To address these gaps, we hypothesized that a synergistic integration of WGCNA and machine learning could uncover prognostic signatures mechanistically linked to tumor-immune interplay. Leveraging multi-center cohorts (TCGA, GEO, METABRIC) and single-cell transcriptomics, this study aimed to: 1. Develop a hybrid AI framework (WGCNA-StepCox-RSF) for robust prognostic modeling; 2. Decipher immune landscape remodeling governed by signature genes; 3. Experimentally validate candidate drivers of tumor progression and immune evasion.

Our work represents a paradigm shift from static prognostic models to dynamic biomarkers interrogating tumor-TME interdependencies. By functionally connecting EGR3 to both tumor suppression and cytotoxic T cell activation, we provide actionable insights for overcoming immunotherapy resistance—a critical unmet need in breast cancer management.

## 2 Methods and materials

### 2.1 Data acquisition and preprocessing

Datasets: Publicly available transcriptomic and clinical data were obtained from The Cancer Genome Atlas (TCGA-BRCA, n=1,098 tumor/113 normal) and Gene Expression Omnibus (GSE42568: 121 tumor/17 normal; GSE9893: 155 tumor; GSE7390: 198 tumor). The METABRIC cohort (n=1,904) served as an independent validation set. Raw RNA-seq and microarray data were normalized using DESeq2 (TCGA) and limma (GEO), with batch effects corrected via ComBat. Clinical endpoints included overall survival (OS) and relapse-free survival (RFS).

Batch Effect Correction: To address technical variations across multiple datasets, we performed systematic batch effect correction. All datasets were individually normalized using z-score scaling, followed by identification of common genes across platforms. Batch effects were removed using the ComBat algorithm from the sva R package, with each dataset treated as a separate batch. Correction effectiveness was validated through principal component analysis (**Supplementary Figure S1A**). All subsequent analyses were conducted using the batch-corrected data.

Inclusion Criteria: (1) Histologically confirmed breast cancer; (2) Complete survival data; (3) RNA sequencing/microarray available.

### 2.2 Differential expression and WGCNA analysis

Differential Expression Analysis: DEGs between tumor and normal tissues were first identified, utilizing DESeq2 (|log2FC| > 1, FDR < 0.05) for the TCGA cohort and limma (adj. p < 0.05) for the GEO dataset. Subsequently, these DEGs were subjected to Weighted Gene Co-expression Network Analysis (WGCNA) to identify clinically relevant modules. Using a soft-thresholding power of β = 6 to achieve a scale-free topology (R² > 0.85), we constructed co-expression networks and identified gene modules via dynamic tree cutting. The turquoise and blue modules, which showed the strongest correlations with clinical outcomes, were prioritized. Hub genes were rigorously defined within these modules by applying dual thresholds of module membership (MM) > 0.8 and gene significance (GS) > 0.2, culminating in the selection of 102 high-confidence hub genes for the construction of the prognostic model.

### 2.3 Machine learning-based prognostic modeling

Feature Selection: The intersection of DEGs and WGCNA hub genes yielded 102 candidate genes, which were further refined to 51 genes shared across all cohorts. Algorithm Optimization: Six machine learning methods (StepCox, LASSO, Ridge, Elastic Net, RSF, SVM) were evaluated using 10-fold cross-validation. The StepCox-RSF hybrid model was ultimately selected based on its superior performance in integrated Brier scores and C-index. Random Survival Forest (RSF) Modeling: The RSF model was implemented using the randomForestSRC R package. Key hyperparameters, including the number of trees (ntree = 1000), the minimum node size (nodesize = 5), and the number of variables tried at each split (mtry = 2), were optimized via 10-fold cross-validation with the goal of minimizing the C-index error. Variable importance (VIMP) and minimal depth analysis were then applied to the optimized RSF model to identify the final 3-gene signature. Model performance was rigorously assessed using time-dependent ROC analysis (timeROC package) and Kaplan-Meier survival analysis.

### 2.4 Multi-cohort validation and immune profiling

Validation Cohorts: Prognostic scores were calculated as: Score = Σ(Genei Expression × RSF Coefficienti) Risk stratification used median cutoffs. Immune Microenvironment Analysis: CIBERSORT: Estimated immune cell fractions from bulk RNA-seq (LM22 signature matrix). ESTIMATE: Computed stromal/immune scores using estimate R package. Checkpoint Molecules: PD-L1 (CD274), LAG3, TIGIT expression quantified via normalized TPM/RSEM. Functional Enrichment: DEGs between subtypes underwent GO and KEGG analysis using clusterProfiler (FDR <0.05).

### 2.5 Single-cell RNA sequencing and functional validation

Single-Cell Data Processing: Public scRNA-seq datasets (GSE176078, GSE161529) were analyzed using Seurat (v4.0). Cells were clustered (resolution=0.8) and annotated via marker genes. Gene expression patterns were visualized using UMAP.

### 2.6 Cell culture

Murine breast cancer cell line 4T1 (American Type Culture Collection, ATCC), were maintained in Dulbecco’s Modified Eagle Medium (DMEM; Gibco) supplemented with 10% fetal bovine serum (FBS; HyClone) and 1% penicillin-streptomycin (Sigma-Aldrich). Cells were incubated at 37 °C in a humidified atmosphere containing 5% CO_2_ and passaged at 80–90% confluence using 0.25% trypsin-EDTA (Gibco). Cell line authenticity was verified by short tandem repeat (STR) profiling, and mycoplasma contamination was routinely tested using a MycoAlert Kit (Lonza).

### 2.7 EGR3 overexpression

For stable EGR3 overexpression, lentiviral particles were generated by co-transfecting HEK293T cells with the pLVX-EGR3 plasmid (Clontech) and packaging plasmids (psPAX2 and pMD2.G) using Lipofectamine 3000 (Invitrogen). Viral supernatants were harvested at 48 and 72 hours post-transfection, concentrated via centrifugation (50,000 × *g*, 4 °C, 2 hours), and titrated using a Lenti-X qRT-PCR Titration Kit (Takara). Target cells (4T1) were transduced at a multiplicity of infection (MOI) of 10 in the presence of 8 μg/mL polybrene (Sigma-Aldrich). Stable clones were selected with 2 μg/mL puromycin (InvivoGen) for 7 days, and overexpression efficiency was confirmed by quantitative RT-PCR and Western blotting.

### 2.8 Cell proliferation

Cells (2 × 10³/well) were seeded in 96-well plates. Proliferation was assessed at 48 and 72 hours using a Cell Counting Kit-8 (CCK-8; Dojindo) following the manufacturer’s protocol. Briefly, 10 μL CCK-8 reagent was added to each well, incubated for 2 hours at 37 °C, and absorbance was measured at 450 nm using a microplate reader (BioTek).

### 2.9 Migration assay

Cell migration was evaluated using Transwell chambers (Corning) with 8-μm pores. Serum-starved cells (5 × 10^4^ in 200 μL serum-free DMEM) were added to the upper chamber, while the lower chamber contained DMEM with 20% FBS as a chemoattractant. After 24 hours, non-migrated cells were removed with a cotton swab. Migrated cells on the lower membrane surface were fixed with 4% paraformaldehyde, stained with 0.1% crystal violet, and imaged under a light microscope (Nikon Eclipse). Cell numbers were quantified using ImageJ software (NIH).

### 2.10 *In vivo* xenograft studies

All animal procedures were approved by the Institutional Animal Care and Use Committee (IACUC) of Chongqing Medical university and conducted in compliance with ARRIVE guidelines. Female BALB/c mice (6–8 weeks old; Charles River Laboratories) were randomized into two groups (*n* = 6/group). For tumor induction, 4T1 cells (1 × 10^6^ cells in 100 μL PBS) transfected with EGR3 or empty vector were injected into the fourth mammary fat pad. Tumor dimensions (length and width) were measured every 3 days using calipers, and tumor volume was calculated as: Volume=0.5×length×width^2^. Mice were euthanized at day 21 post-injection, and tumors were excised, weighed, and processed for downstream analyses.

### 2.11 Isolation of human peripheral blood mononuclear cells

Peripheral blood samples were collected from healthy adult volunteers under an institution-approved protocol. PBMCs were isolated using Ficoll-Paque PLUS (Cytiva) density gradient centrifugation according to the manufacturer’s instructions. Briefly, fresh blood was diluted 1:1 with phosphate-buffered saline (PBS). The diluted blood was carefully layered over an equal volume of Ficoll-Paque solution and centrifuged at 400 × g for 30 minutes at room temperature with the brake off. The PBMC layer at the interface was carefully aspirated, washed twice with PBS, and resuspended in complete RPMI-1640 medium supplemented with 10% fetal bovine serum (FBS) and 1% penicillin-streptomycin. Cell viability, assessed by Trypan Blue exclusion, was consistently >95%.

### 2.12 *In vitro* co-culture of tumor cells and PBMCs

To investigate the immunomodulatory effect of EGR3-expressing tumor cells on T cell function within a more physiologically relevant immune context, we established a co-culture system using human PBMCs. Control (Vector) and EGR3-overexpressing (EGR3-OE) MDA-MB-231 human triple-negative breast cancer cells were seeded in 12-well plates and allowed to adhere overnight. The following day, freshly isolated PBMCs from healthy donors were added to the tumor cells at a tumor cell to PBMC ratio of 1:10. The co-culture was maintained in complete RPMI-1640 medium for 48 hours. PBMCs cultured alone in complete medium served as a baseline control. To evaluate T cell cytokine production, brefeldin A (1:1000, BioLegend) was added to the culture for the final 4–6 hours to inhibit protein transport, allowing for intracellular accumulation of cytokines. After co-culture, cells were harvested, stained for surface markers (CD8), fixed, permeabilized, and then stained intracellularly for IFN-γ and TNF-α prior to flow cytometry analysis of CD8^+^ T cell function.

### 2.13 Flow cytometry

Tumors were minced and enzymatically dissociated into single-cell suspensions using a Tumor Dissociation Kit (Miltenyi Biotec) and the gentleMACS Octo Dissociator (Miltenyi Biotec). Red blood cells were lysed with ACK buffer (Gibco). Cells were stained with fluorochrome-conjugated antibodies against CD8a (APC, clone 53-6.7), IFN-γ (PE, clone XMG1.2), and TNF-α (FITC, clone MP6-XT22; all from BioLegend) for 30 minutes at 4 °C in the dark. After washing, data were acquired on a BD FACSymphony™ flow cytometer (BD Biosciences) and analyzed using FlowJo v10.8 software (TreeStar). Gating strategies excluded doublets and dead cells using 7-AAD viability dye.

### 2.14 Statistical analysis

Data from at least three independent experiments are presented as mean ± SEM. Statistical significance was determined using two-tailed Student’s t-tests for comparisons between two groups. For comparisons across multiple groups, one-way ANOVA was performed, followed by Tukey’s honest significant difference (HSD) test for *post-hoc* analysis. In the survival analysis, the Benjamini-Hochberg procedure was applied to control the false discovery rate (FDR) for multiple comparisons across genes. All statistical tests were two-sided, and a p-value of less than 0.05 was considered statistically significant. Analyses were performed using GraphPad Prism software (version 9.0).

## 3 Results

### 3.1 WGCNA-based discovery of prognostic biomarkers in breast cancer

**Figure 1:**
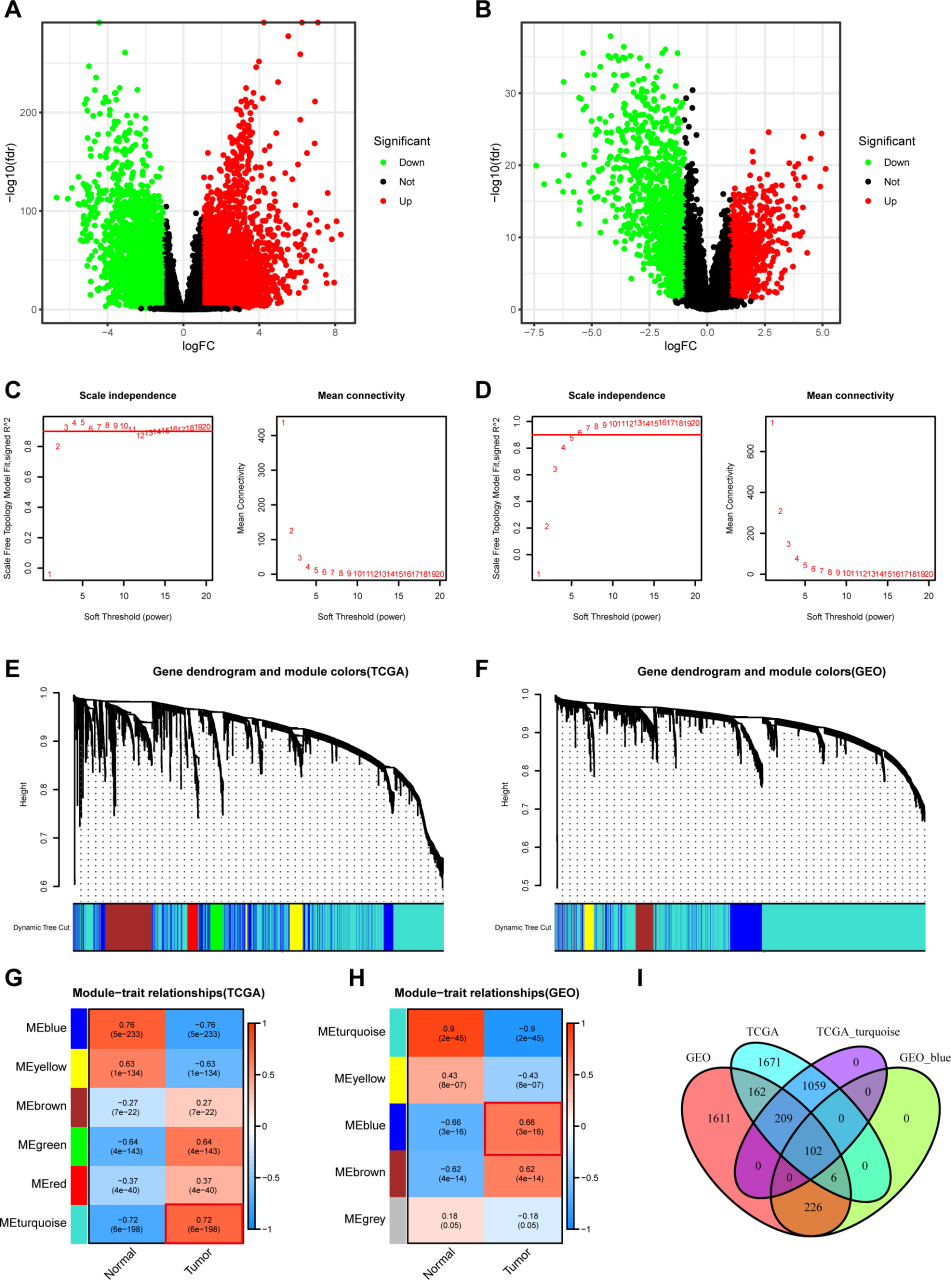
Identification of prognosis-related genes in breast cancer based on WGCNA. **(A, B)** Volcano plots of differentially expressed genes between breast cancer and normal tissues in TCGA and GSE42568 datasets. **(C, D)** Visualization of thresholded genes from WGCNA analysis in TCGA and GSE42568 datasets. **(E, F)** Clustering dendrogram from WGCNA analysis of TCGA and GSE42568 datasets. **(G, H)** Presentation of WGCNA-derived co-expression modules distinguishing tumor-normal samples in TCGA and GEO (GSE42568) cohorts. **(I)** Venn diagram illustrating gene overlaps between differential expression analysis and key co-expression modules.

To identify key genes involved in the pathogenesis and progression of breast cancer, we performed differential expression analysis between tumor and normal samples using datasets from TCGA and GSE42568 databases. As shown in **Figures 1A, B**, thousands of differentially expressed genes (DEGs) were identified in both datasets. To refine these candidate genes, we subsequently employed Weighted Gene Co-expression Network Analysis (WGCNA) to identify prognosis-related key module genes (**Figures 1C–F**). The results demonstrated that the turquoise module showed the strongest tumor correlation in the TCGA dataset, while the blue module exhibited the highest tumor association in the GSE42568 dataset (**Figures 1G, H**). Finally, through intersection analysis of DEGs from TCGA, GEO datasets, along with genes from the turquoise and blue modules identified by WGCNA, we identified 102 hub genes (**Figure 1I**). These findings suggest that these 102 hub genes may play crucial roles in breast cancer progression.

### 3.2 Machine learning-based screening of optimal modeling approaches for prognostic gene signatures

**Figure 2:**
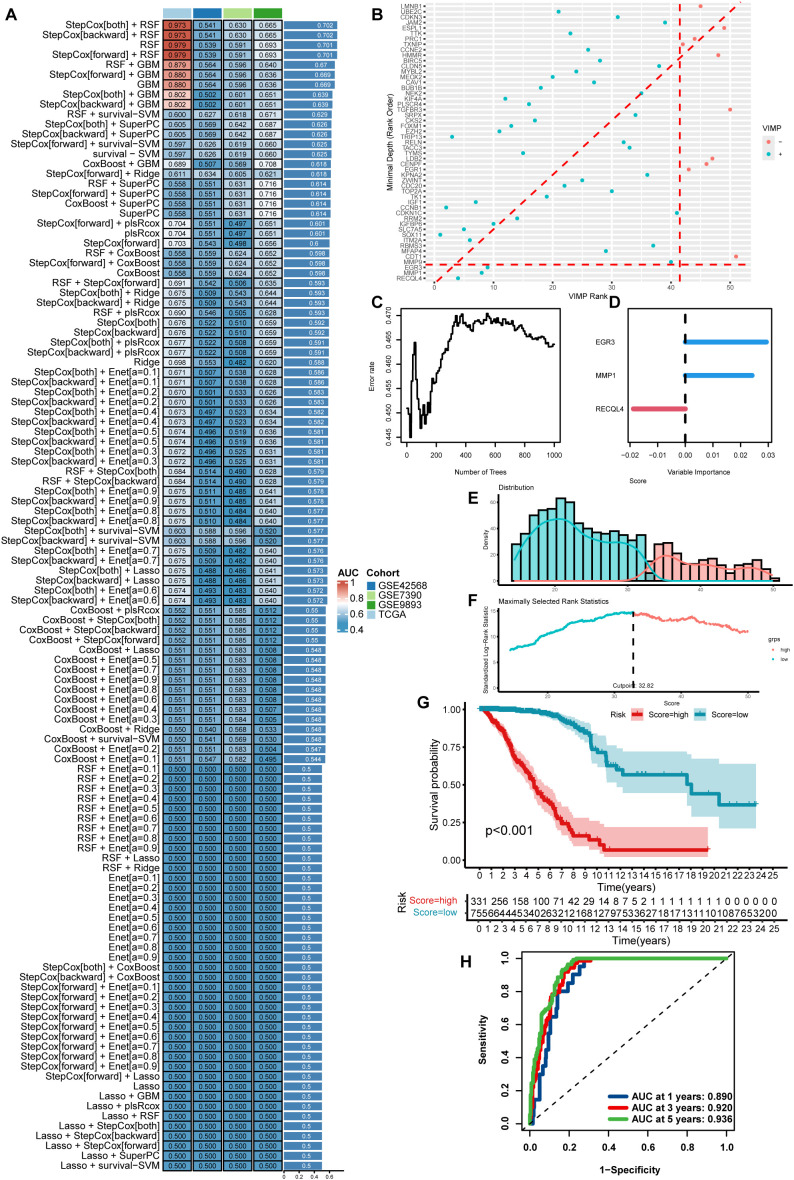
Construction of a prognostic signature for prognosis-related genes based on machine learning. **(A)** Machine learning-based optimization of predictive modeling across four breast cancer datasets. **(B)** VIMP ranking identifies top contributory genes in the predictive model. **(C, D)** Evaluation of model stability (error tolerance rate) with VIMP measures for critical genes. **(E, F)** Random survival forest (RSF)-based modeling of survival distribution across classified patient subgroups. **(G)** Subtype-specific survival analysis in breast cancer patients. **(H)** Time-dependent ROC analysis evaluating predictive performance of RSF-derived subtype classification.

To identify an optimal approach for constructing prognostic models, we expanded our analysis by incorporating additional datasets including GSE9893 and GSE7390 alongside previous cohorts. Machine learning analysis was performed on 51 shared genes across these datasets (**Supplementary Figures S1B, C**). Comparative evaluation revealed that the StepCox method combined with Random Survival Forest (RSF) demonstrated superior performance in prognostic model construction (**Figure 2A**). Subsequently, RSF modeling was applied to these 51 genes for prognostic prediction. Through VIMP ranking and minimal depth analysis, we identified a 3-gene prognostic signature (**Figure 2B**), with corresponding error rate validation (**Figure 2C**) and VIMP visualization (**Figure 2D**).

The prognostic model based on these three genes exhibited significant survival discrimination (**Figures 2E, F**). Patients stratified into the high-risk group showed markedly worse clinical outcomes (**Figure 2G**). Time-dependent ROC analysis revealed robust predictive accuracy, with AUC values of 0.890, 0.920, and 0.936 for 1-, 3-, and 5-year survival predictions, respectively (**Figure 2H**). These results demonstrate that the machine learning-optimized model integrating StepCox and RSF methodologies provides enhanced prognostic stratification capacity for breast cancer patients.

### 3.3 Development and multi-cohort validation of an RSF-based prognostic signature

**Figure 3:**
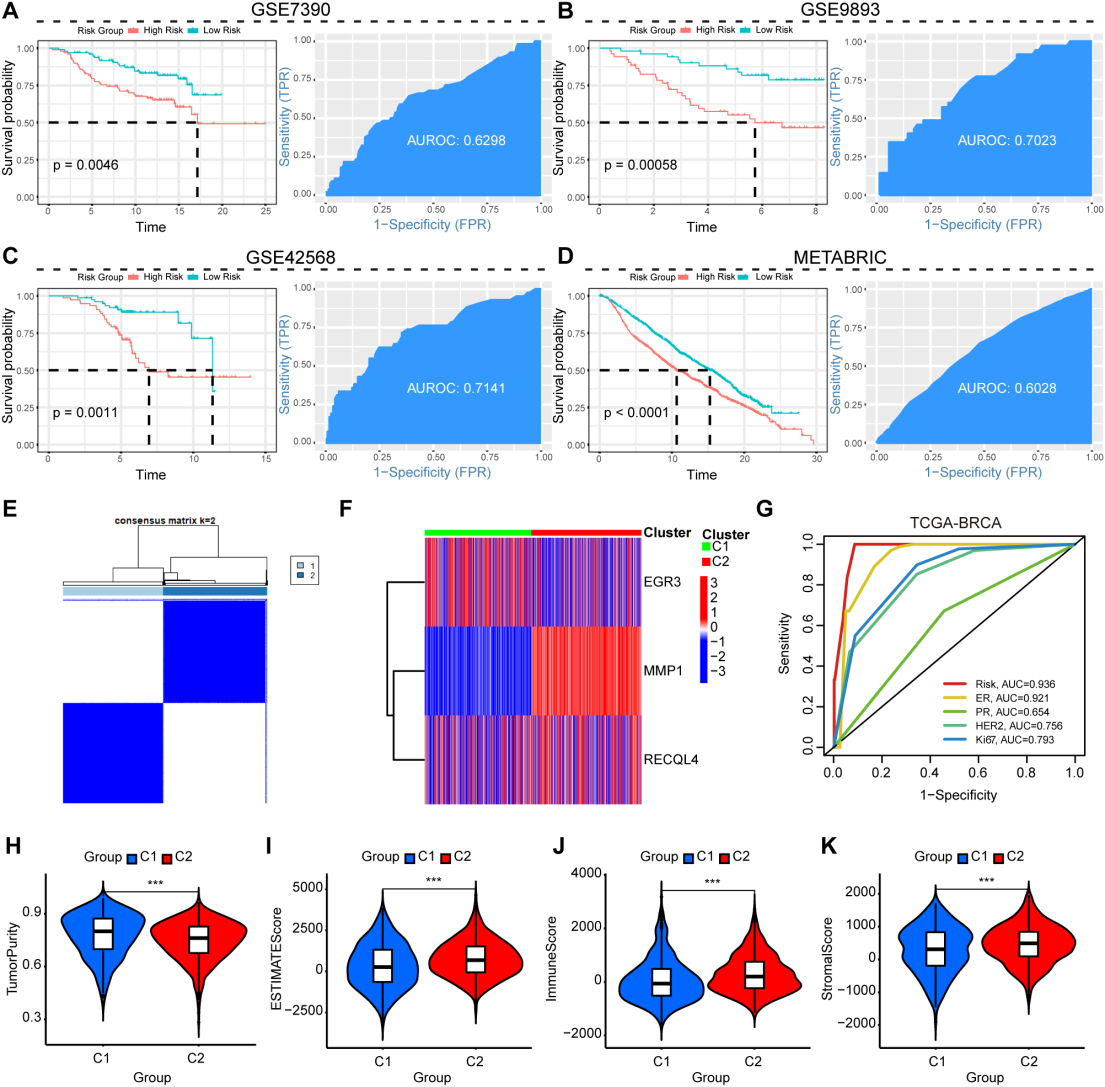
Clustering of breast cancer patients based on prognostic signature genes. **(A–D)** Validation of RSF-derived survival analysis and predictive ROC curves using GSE7390, GSE9893, GSE42568, and METABRIC datasets as independent cohorts. **(E)** Stratification of breast cancer patients into two subgroups based on prognostic signature genes. **(F)** Differential expression visualization of prognostic signature genes in dichotomized breast cancer subgroups. **(G)** Time-dependent ROC curves comparing the prognostic accuracy of the three-gene signature combined with standard clinical variables (ER, PR, HER2, and Ki67 status) versus clinical variables alone for 5-year survival in the full cohort. **(H–K)** Violin plots illustrating relationships between dichotomous breast cancer subtypes and tumor purity, ESTIMATE scores, immune scores, and stromal scores. ***P<0.001.

The validation cohorts (GSE7390, GSE9893, GSE42568, and METABRIC) were utilized to assess the prognostic performance of the model. Results demonstrated that breast cancer patients with higher prognostic signature scores exhibited significantly worse clinical outcomes (**Figures 3A–D**). Time-dependent ROC analysis revealed area under the curve (AUC) values of 0.6298 for GSE7390, 0.7023 for GSE9893, 0.7141 for GSE42568, and 0.6028 for METABRIC, further confirming the model’s predictive reliability across independent datasets (**Figures 3A–D**). Further stratification based on expression levels of the three model-constructing genes (EGR3, RECQL4, and MMP1) revealed two distinct breast cancer subtypes (**Figure 3E**), with significant inter-subtype expression differences visualized in **Figure 3F**. The integrated model combining the three-gene signature with key clinical variables (ER, PR, HER2, and Ki67 status) demonstrated a superior prognostic ability compared to the model with clinical variables alone, as evidenced by higher AUC values across 5-year survival predictions (**Figure 3G**). ESTIMATE algorithm-based analysis demonstrated that patients in the C1 subtype exhibited significantly higher tumor purity (**Figure 3H**), along with reduced ESTIMATE scores (**Figure 3I**), lower immune infiltration (**Figure 3J**), and diminished stromal component (**Figure 3K**) compared to other subgroups.

### 3.4 Immune landscape characterization of prognostic gene signature-defined breast cancer subtypes

**Figure 4:**
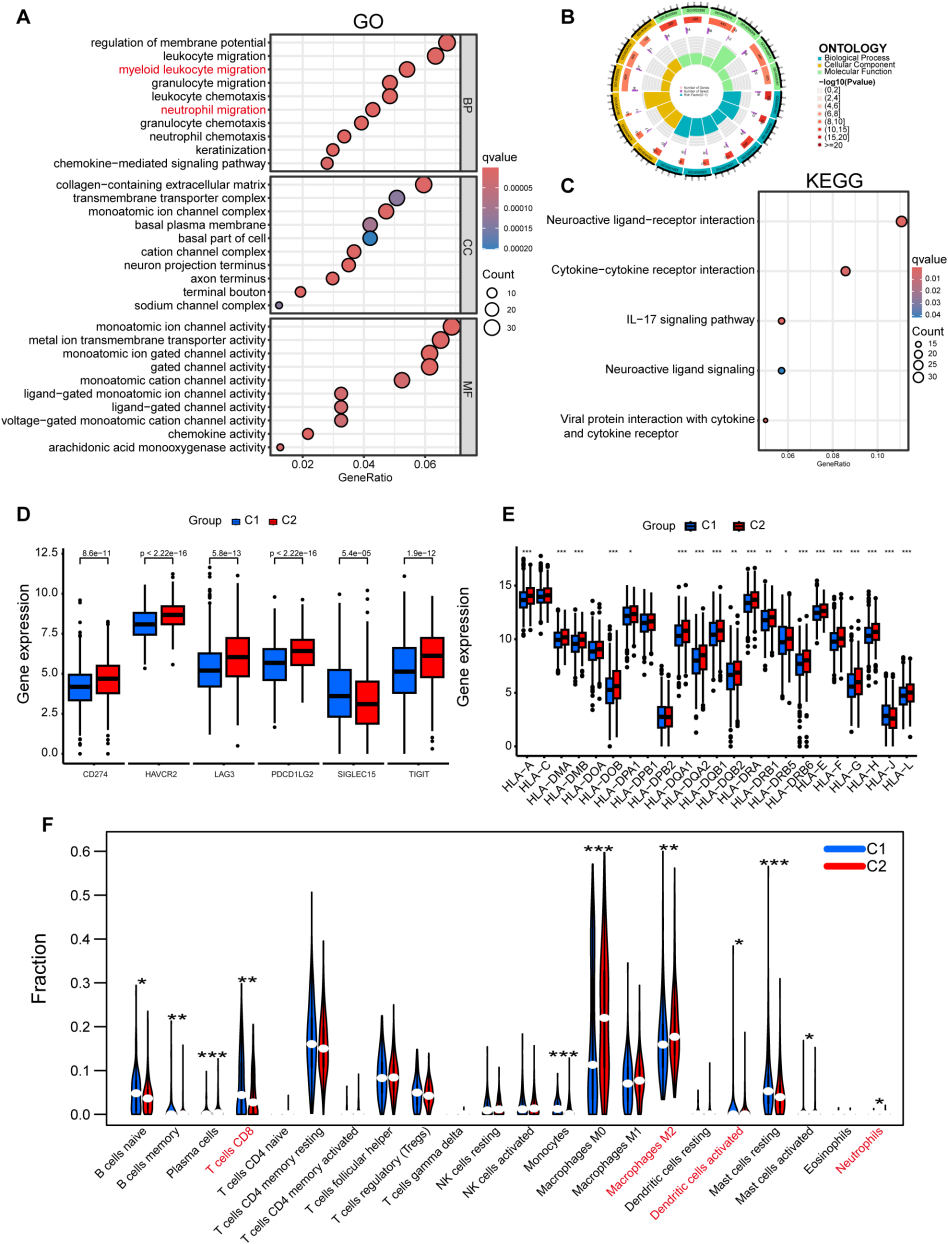
The prognostic signature stratifies distinct breast cancer subtypes and is associated with immune infiltration. **(A–C)** GO and KEGG enrichment analyses reveal differential genes associated with myeloid immune cells and IL-17 signaling pathway across classified breast cancer subtypes. **(D)** Differential expression patterns of immune checkpoint regulators across breast cancer molecular subtypes. **(E)** Relationship of breast cancer subtypes with HLA family genes. **(F)** Immune infiltration profiling of RSF-classified breast cancer subtypes using CIBERSORT algorithm: Comparative analysis of immune cell composition. *P<0.05, **P<0.01, ***P<0.001.

To investigate the mechanistic basis of the three-gene prognostic signature (EGR3, RECQL4, MMP1) in breast cancer progression, we performed differential expression analysis followed by GO and KEGG pathway enrichment analyses between the two molecular subtypes. Functional annotation revealed significant enrichment of immune-related pathways, including myeloid cell migration, neutrophil chemotaxis, and IL-17 signaling pathway among differentially expressed genes (**Figures 4A–C**).

To validate the immunological relevance of the prognostic model, we systematically profiled immune-related molecules across subtypes. The C2 subtype exhibited elevated expression of immune checkpoint inhibitors, including CD274 (PD-L1), LAG3, and TIGIT (**Figure 4D**), along with upregulated HLA family genes critical for antigen presentation (**Figure 4E**). Notably, CIBERSORT deconvolution of TCGA breast cancer samples demonstrated an immunosuppressive microenvironment in the C2 subgroup, characterized by reduced CD8^+^ T cell infiltration, enriched M2 macrophage polarization, and expanded neutrophil populations (**Figure 4F**). These findings collectively establish that our prognostic signature is mechanistically linked to tumor immunomodulation.

### 3.5 Clinical relevance of prognostic gene signatures and functional characterization of EGR3 in breast cancer

**Figure 5:**
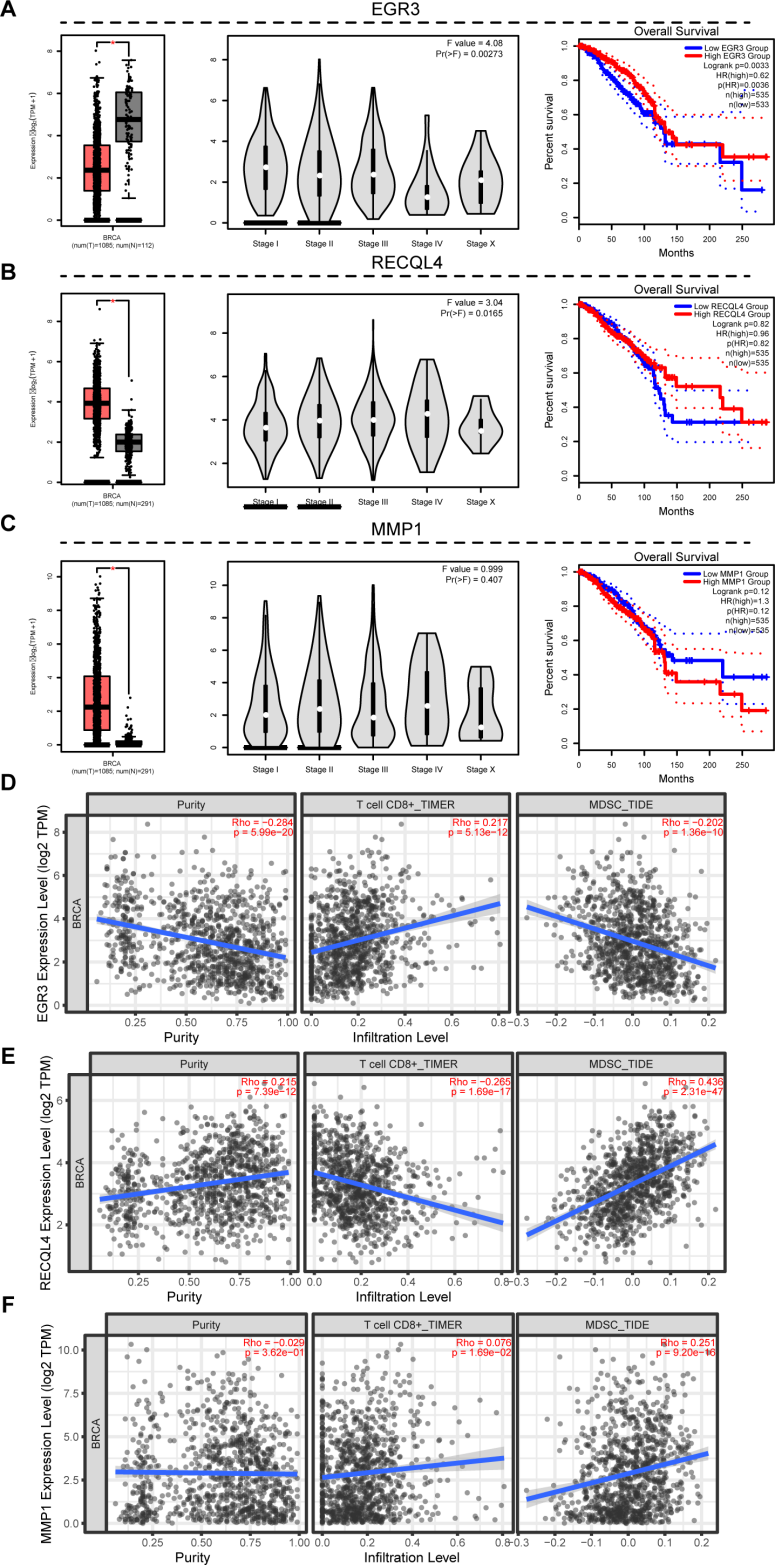
Prognostic signature genes are associated with immune infiltration. **(A–C)** Comprehensive analysis of RSF prognostic signature genes (EGR3, RECQL4, MMP1) in TCGA: Tumor-normal differential expression; stage-specific expression patterns; and survival outcomes. **(D–F)** Correlation analysis between RSF prognostic signature genes (EGR3, RECQL4, MMP1) and tumor-infiltrating CD8^+^ T cells/MDSCs.

**Figure 6:**
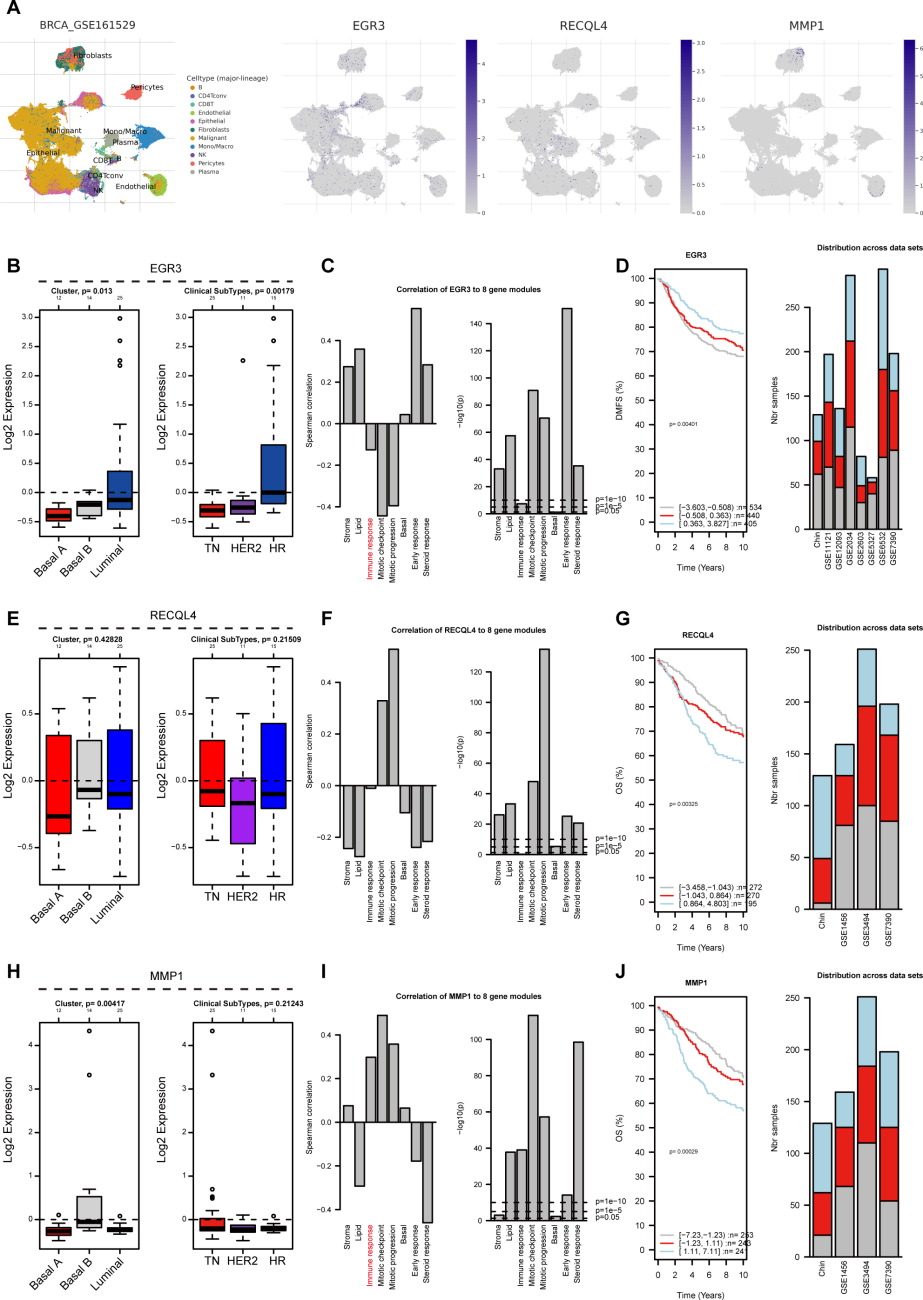
The relationship between prognostic signature genes and clinical characteristics of breast cancer patients. **(A)** Single-cell transcriptomic mapping of RSF-derived prognostic genes (EGR3/RECQL4/MMP1) using UMAP dimensionality reduction in the GSE161529 cohort. **(B, E, H)** Differential expression of EGR3, RECQL4, and MMP1 across PAM50 subtypes and molecular classifications in breast cancer. **(C, F, I)** Correlation analysis between EGR3/RECQL4/MMP1 and distinct gene modules. **(D, G, J)** Prognostic analysis of EGR3, RECQL4, and MMP1 across multiple independent cohorts.

To elucidate the functional roles of the three prognostic signature genes (EGR3, RECQL4, and MMP1) in breast cancer pathogenesis, we conducted comprehensive multi-omics analyses across multiple datasets. Pan-cancer expression profiling revealed significant downregulation of EGR3 in tumor tissues compared to normal counterparts, with progressive reduction across advancing tumor stages. Survival analysis demonstrated favorable clinical outcomes in EGR3-high patients, contrasting with the oncogenic patterns of RECQL4 and MMP1, though their survival correlations lacked statistical significance (**Figures 5A–C**). Immune infiltration analysis revealed EGR3 exhibited inverse correlation with tumor purity, positive association with CD8^+^ T cell infiltration, and negative correlation with myeloid-derived suppressor cells (MDSCs). Conversely, RECQL4 and MMP1 displayed opposing immunomodulatory patterns (**Figures 5D–F**). Single-cell RNA sequencing across tumor microenvironment compartments demonstrated ubiquitous but cell type-specific expression patterns, with notable enrichment in malignant cells observed in select datasets (**Supplementary Figures S2A–C**; **Figure 6A**). Subtype-specific evaluation revealed lowest EGR3 expression in triple-negative breast cancer (TNBC), correlating strongly with immune response signatures. Multi-cohort validation confirmed EGR3-high tumors exhibited superior prognosis (**Figures 6B–D**; **Supplementary Figure S3A**). In contrast, RECQL4 and MMP1 showed minimal subtype-specific variation and immune association, though differential survival patterns emerged (**Figures 6E–J**; **Supplementary Figures S3B, C**). Cell line validation confirmed TNBC-specific EGR3 suppression (**Supplementary Figures S3D–F**).

**Figure 7:**
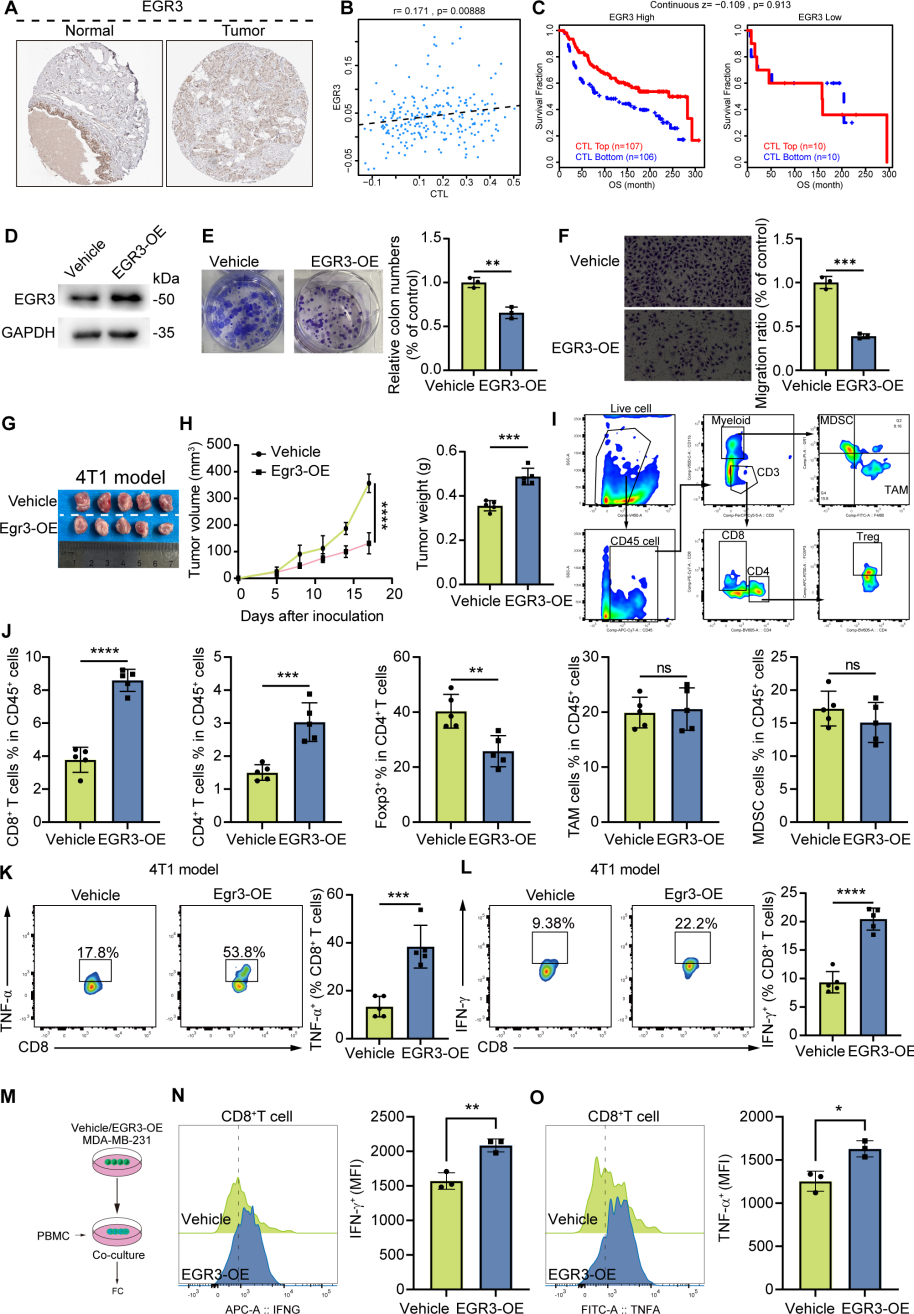
EGR3 suppresses MDSC infiltration in breast cancer and enhances anti-tumor immunity. **(A)** Immunohistochemical (IHC) analysis of EGR3 expression in breast cancer versus normal breast tissues. **(B)** Correlation between EGR3 and cytotoxic T lymphocytes (CTL). **(C)** Impact of EGR3 expression and cytotoxic T lymphocyte (CTL) infiltration on breast cancer prognosis. **(D)** Western blot (WB) analysis of EGR3 overexpression effects. **(E)** CCK-8 assay validation of EGR3 overexpression effects on breast cancer cell proliferation. **(F)** Transwell migration assay assessing EGR3 overexpression effects on breast cancer cell motility. **(G)** Subcutaneous xenograft model demonstrating the impact of EGR3 overexpression on breast cancer growth *in vivo*. **(H)** Comparative analysis of subcutaneous tumor growth kinetics and endpoint tumor weights between EGR3-overexpressing and control groups (n=5). **(I)** Flow cytometry gating strategy visualization. **(J)** Impact of EGR3 overexpression on tumor microenvironment (TME) cellular composition in breast cancer. **(K, L)** Effects of EGR3 overexpression on CD8^+^ T cell function. **(M)** Schematic of the tumor cellrPBMCs co-culture workflow. **(N, O)** Flow-cytometric analysis of functional T-cell markers after co-culture. ****P<0.0001, ***P<0.001, **P<0.01, *P<0.05.

Prioritizing EGR3 for mechanistic validation, clinical specimen analysis confirmed significant EGR3 downregulation in breast carcinomas (**Figure 7A**). TIDE database interrogation revealed positive correlation between EGR3 expression and cytotoxic T lymphocyte (CTL) infiltration (**Figure 7B**), with EGR3-high cohorts demonstrating enhanced immunotherapeutic response (**Figure 7C**). Furthermore, EGR3 expression was significantly correlated with the levels of multiple immune-related molecules, including LAG3, CD274 (PD-L1), TIGIT, and CD69 (**Supplementary Figure S4**). Functional studies in 4T1 murine models demonstrated EGR3 overexpression significantly inhibited tumor proliferation and suppressed migratory capacity (**Figures 7D–F**). *In vivo* xenograft experiments revealed 56.7% tumor volume reduction with EGR3 overexpression (**Figures 7G, H**). Flow cytometric analysis of tumor-infiltrating lymphocytes demonstrated enhanced CD8^+^ T cell activation and increased TNF-α and IFN-γ production in EGR3-overexpressing tumors (**Figures 7I–L**). These findings suggest an association between EGR3 expression and tumor-suppressive immunomodulatory effects with therapeutic potential in breast cancer. To determine if EGR3 expression directly augments T cell effector function, we performed a tumor cell-PBMC co-culture assay. We found that EGR3-overexpressing tumor cells significantly boosted the proportion of cytokine-producing CD8^+^ T cells, indicating a potent role for EGR3 in promoting T cell activation (**Figures 7M–O**).

## 4 Discussion

The advent of immune checkpoint inhibitors (ICIs) has revolutionized cancer treatment, yet their efficacy in breast cancer remains limited to subsets of triple-negative breast cancer (TNBC) with PD-L1 positivity or high tumor-infiltrating lymphocytes (TILs) (9). Despite FDA approvals for pembrolizumab in PD-L1+ metastatic TNBC, objective response rates stagnate at 18-35%, with acquired resistance emerging as a critical barrier (10). This therapeutic plateau underscores the urgent need for biomarkers that transcend conventional PD-L1 assessment by interrogating dynamic tumor-immune interactions. Current strategies predominantly focus on single-dimensional biomarkers—including PD-L1 immunohistochemistry, tumor mutational burden (TMB), and TIL quantification—which exhibit variable predictive power across molecular subtypes and treatment lines (11). For instance, while KEYNOTE-355 (12) validated PD-L1 Combined Positive Score (CPS ≥10) as a predictive marker, 40% of PD-L1+ patients still show primary resistance, highlighting the complexity of immunosuppressive mechanisms beyond checkpoint ligand expression.

Recent advances implicate MDSCs, tumor-associated macrophages (TAMs), and interleukin signaling in shaping breast cancer immunogenicity (13). However, existing prognostic models rarely integrate these immunological dimensions with genomic features, creating a disconnect between risk stratification and therapeutic actionability. The IMpassion130 trial first demonstrated the clinical relevance of combining immune gene signatures (e.g., IFN-γ) with angiogenesis markers, yet such approaches lack mechanistic links to tumor-intrinsic pathways (14). Meanwhile, emerging machine learning studies attempt to decode tumor-immune crosstalk through multi-omics integration, but most remain confined to computational predictions without experimental validation. This gap between algorithmic discovery and biological validation hinders the development of biomarkers capable of guiding combination therapies targeting both tumor cells and immunosuppressive niches.

This study establishes a machine learning-optimized 3-gene prognostic signature (EGR3, RECQL4, MMP1) that bridges prognostic stratification and tumor-immune interplay in breast cancer, offering both clinical and mechanistic advancements. Our integrative approach, combining WGCNA, multi-cohort survival analytics, and functional validation, addresses critical gaps in current biomarker research while providing novel insights into immune microenvironment regulation. Below, we contextualize our findings within the evolving landscape of cancer immunology and precision oncology, highlighting how this work extends beyond existing paradigms.

The synergistic integration of WGCNA and machine learning represents a methodological leap (15). While WGCNA has been widely adopted for module identification in cancer studies, most prior works halted at candidate gene selection without clinical model optimization (16). By contrast, our StepCox-RSF framework advanced this pipeline by rigorously screening 51 candidates to a 3-gene signature with cross-platform validity (AUC >0.89 across four cohorts), demonstrating superior performance to conventional Cox model. This aligns with emerging trends in computational oncology where ensemble algorithms (e.g., RSF, XGBoost) are increasingly favored for handling high-dimensional omics data (17), yet our study uniquely validates such approaches in breast cancer across diverse ethnic and technical platforms (TCGA, METABRIC).

The immunological dimension of our prognostic model distinguishes it from existing signatures. Current immune-based biomarkers predominantly focus on checkpoint inhibitors (e.g., PD-L1) or tumor mutational burden, which exhibit variable predictive utility across subtypes (18). Our work reveals that the 3-gene signature stratifies patients into immunologically distinct subgroups: C2 tumors displayed concurrent PD-L1 elevation and CD8^+^ T cell depletion, a paradoxical “immune-hot but dysfunctional” phenotype increasingly recognized in immunotherapy-resistant cohorts (19). This contrasts with traditional immune classifications by linking prognostic outcomes to myeloid cell chemotaxis and IL-17 signaling, pathways recently implicated in breast cancer immune evasion (20). Importantly, EGR3 emerged as a potential immunomodulator, showing association with cytotoxic lymphocyte infiltration and immunotherapy response—a finding that expands upon previous reports of EGR3’s role in T cell activation and suggests its relevance as a breast-specific therapeutic candidate.

The RSF algorithm’s capacity to capture non-linear relationships and gene interactions proved pivotal in refining the prognostic signature (21). Traditional methods like LASSO-Cox often oversimplify complex biological systems by assuming linear additive effects (22), whereas RSF’s VIMP ranking revealed RECQL4’s non-linear association with survival—a pattern undetectable by conventional regression. This aligns with recent studies demonstrating machine learning’s superiority in modeling tumor-immune crosstalk (23), yet our work extends these principles by validating model robustness across single-cell and xenograft datasets. Furthermore, the time-dependent AUC values exceeding 0.93 at 5 years surpass those of published signatures, underscoring the translational potential of algorithm-optimized models.

EGR3: While recognized as a stress-response transcription factor in neurodegeneration, its role in cancer remains contentious (24). Prior pan-cancer analyses noted EGR3’s tumor-suppressive effects in gliomas (25) but oncogenic functions in lung adenocarcinoma (26). Our findings resolve this discrepancy by demonstrating context-dependent roles: In breast cancer, EGR3 downregulation correlates with advanced stages and immunosuppression, while its overexpression inhibits proliferation and enhances CTL activity. This observed association with both cell-intrinsic growth regulation and immune activation—contrasts with single-mechanism biomarkers like PD-L1 and warrants further investigation of EGR3 as a potential therapeutic target. RECQL4: Best known for its DNA helicase activity in genome maintenance, RECQL4 has been implicated in chemotherapy resistance (27). Our immune correlation analysis uncovered its novel association with MDSC infiltration, suggesting a non-canonical role in myeloid-mediated immunosuppression. This aligns with recent work linking RECQL4 to CXCL12 secretion in osteosarcoma but provides the first evidence of its immunomodulatory function in breast cancer. MMP1: Although MMPs are classically associated with extracellular matrix remodeling (28), our single-cell analysis revealed malignant cell-specific MMP1 enrichment, implicating tumor-intrinsic signaling in metastasis beyond stromal interactions. This challenges the prevailing stromal-centric view of MMP1 and suggests autocrine regulatory mechanisms warranting further study.

While direct comparative analysis with established commercial signatures such as PAM50, Oncotype DX, and MammaPrint was beyond the scope of this study, our 3-gene signature demonstrates competitive prognostic performance, with time-dependent AUC values exceeding 0.93 at 5 years in the training cohort. However, we acknowledge that comprehensive benchmarking against these clinically validated signatures in matched patient cohorts would be necessary to fully establish its clinical utility. Future studies should focus on head-to-head comparisons in prospective cohorts to determine the added value of our immune-informed signature over existing commercial tests.

Several limitations should be considered when interpreting our findings. While our multi-cohort validation strengthens reliability, retrospective analysis of public datasets may introduce selection bias toward treatment-naïve patients. Additionally, the absence of direct comparison with established commercial signatures (e.g., PAM50, Oncotype DX, MammaPrint) limits our ability to assess the clinical competitive advantage of our 3-gene model. Future validation in cohorts with parallel testing of multiple prognostic signatures will be essential for clinical translation. The signature’s performance in rare subtypes such as metaplastic or inflammatory breast cancer remains unverified due to the scarcity of relevant omics datasets, necessitating future validation in larger, subtype-specific cohorts. Our functional investigation focused on EGR3 via overexpression, as its low endogenous expression in aggressive TNBC models limited the feasibility of knockdown approaches. The precise molecular mechanisms by which EGR3 exerts its immunomodulatory effects, particularly its regulation of cytotoxic T lymphocyte recruitment, therefore require further investigation in models amenable to perturbation of endogenous EGR3. Furthermore, the mechanistic contributions of other signature genes, including RECQL4 and MMP1, to prognosis and immune modulation are not fully defined and warrant dedicated functional studies. Lastly, clinical translation demands prospective trials assessing the signature’s utility in guiding immunotherapy decisions.

## 5 Conclusion

This study redefines prognostic biomarker development through three paradigm shifts: (1) Transition from static gene lists to dynamic immune-interactive signatures; (2) Integration of machine learning with functional genomics to uncover non-linear biomarker relationships; (3) Association of EGR3 with dual-action immunotherapeutic effects. By demonstrating that prognostic models can concurrently predict survival and immunotherapy response, we provide a blueprint for next-generation biomarker discovery. Future efforts should focus on translating the 3-gene signature into clinical assays and exploring EGR3-targeted combination therapies to overcome immune resistance in breast cancer.

## Data Availability

The original contributions presented in the study are included in the article/**Supplementary Material**. Further inquiries can be directed to the corresponding author.

## Supplementary material

Supplementary Figure 1 Display of prognostic-associated genes. **(A)** Visualization of machine learning-relevant genes by VIMP measure. **(B)** STING protein-protein interaction (PPI) network visualization of machine learning-identified genes.

Supplementary Figure 2 Single-cell transcriptional landscape of prognostic signature genes. **(A–C)** Multi-dataset single-cell profiling of EGR3, RECQL4, and MMP1 expression across tumor microenvironment cell types.

Supplementary Figure 3 Prognostic signature genes across different breast cancer subtypes: clinical implications. **(A–C)** Distinct expression patterns of EGR3, RECQL4, and MMP1 across HU classification, PAM50 subtypes, ER status, and histological grades. **(D–F)** Differential expression profiles of EGR3, RECQL4, and MMP1 across breast cancer cell lines.

Supplementary Figure 4 Correlation of EGR3 with immune-related molecules. **(A)** Correlation between EGR3 and LAG3. **(B)** Correlation between EGR3 and CD274. **(C)** Correlation between EGR3 and TIGIT. **(D)** Correlation between EGR3 and CD69.

## References

[1] Xiong X Zheng LW Ding Y Chen YF Cai YW Wang LP . Breast cancer: pathogenesis and treatments. Signal Transduct Target Ther. (2025) 10:49. doi: 10.1038/s41392-024-02108-4, PMID: 39966355 PMC11836418

[2] Jiang YZ Ma D Jin X Xiao Y Yu Y Shi J . Integrated multiomic profiling of breast cancer in the Chinese population reveals patient stratification and therapeutic vulnerabilities. Nat Cancer. (2024) 5:673–90. doi: 10.1038/s43018-024-00725-0, PMID: 38347143

[3] Pradat Y Viot J Yurchenko AA Gunbin K Cerbone L Deloger M . Integrative pan-cancer genomic and transcriptomic analyses of refractory metastatic cancer. Cancer Discov. (2023) 13:1116–43. doi: 10.1158/2159-8290.CD-22-0966, PMID: 36862804 PMC10157368

[4] Li YK Zeng T Guan Y Liu J Liao NC Wang MJ . Validation of ESM1 related to ovarian cancer and the biological function and prognostic significance. Int J Biol Sci. (2023) 19:258–80. doi: 10.7150/ijbs.66839, PMID: 36594088 PMC9760436

[5] Huang D Ma N Li X Gou Y Duan Y Liu B . Advances in single-cell RNA sequencing and its applications in cancer research. J Hematol Oncol. (2023) 16:98. doi: 10.1186/s13045-023-01494-6, PMID: 37612741 PMC10463514

[6] Zhang K Fu R Liu R Su Z . Circulating cell-free DNA-based multi-cancer early detection. Trends Cancer. (2024) 10:161–74. doi: 10.1016/j.trecan.2023.08.010, PMID: 37709615

[7] Cai R Chen Q Zhao D Wang Y Zhou L Zhang K . A high immune-related index with the suppression of cGAS-STING pathway is a key determinant to herceptin resistance in HER2+ Breast cancer. Int J Biol Sci. (2024) 20:3497–514. doi: 10.7150/ijbs.94868, PMID: 38993569 PMC11234227

[8] Zhang J Chen Q Zhang Y Zhou J . Construction of a random survival forest model based on a machine learning algorithm to predict early recurrence after hepatectomy for adult hepatocellular carcinoma. BMC Cancer. (2024) 24(1):1575. doi: 10.1186/s12885-024-13366-4, PMID: 39722042 PMC11670344

[9] Liu Y Hu Y Xue J Li J Yi J Bu J . Advances in immunotherapy for triple-negative breast cancer. Mol Cancer. (2023) 22:145. doi: 10.1186/s12943-023-01850-7, PMID: 37660039 PMC10474743

[10] Geurts V Kok M . Immunotherapy for metastatic triple negative breast cancer: current paradigm and future approaches. Curr Treat Options Oncol. (2023) 24:628–43. doi: 10.1007/s11864-023-01069-0, PMID: 37079257 PMC10172210

[11] Buisseret L Bareche Y Venet D Girard E Gombos A Emonts P . The long and winding road to biomarkers for immunotherapy: a retrospective analysis of samples from patients with triple-negative breast cancer treated with pembrolizumab. ESMO Open. (2024) 9:102964. doi: 10.1016/j.esmoop.2024.102964, PMID: 38703428 PMC11087916

[12] Im SA Cortes J Cescon DW Yusof MM Iwata H Masuda N . Results from the randomized KEYNOTE-355 study of pembrolizumab plus chemotherapy for Asian patients with advanced TNBC. NPJ Breast Cancer. (2024) 10:79. doi: 10.1038/s41523-024-00679-7, PMID: 39266535 PMC11393332

[13] Cassetta L Bruderek K Skrzeczynska-Moncznik J Osiecka O Hu X Rundgren IM . Differential expansion of circulating human MDSC subsets in patients with cancer, infection and inflammation. J Immunother Cancer. (2020) 8(2):e001223. doi: 10.1136/jitc-2020-001223, PMID: 32907925 PMC7481096

[14] Emens LA Adams S Barrios CH Dieras V Iwata H Loi S . First-line atezolizumab plus nab-paclitaxel for unresectable, locally advanced, or metastatic triple-negative breast cancer: IMpassion130 final overall survival analysis. Ann Oncol. (2021) 32:983–93. doi: 10.1016/j.annonc.2021.05.355, PMID: 34272041

[15] Xu M Zhou H Hu P Pan Y Wang S Liu L . Identification and validation of immune and oxidative stress-related diagnostic markers for diabetic nephropathy by WGCNA and machine learning. Front Immunol. (2023) 14:1084531. doi: 10.3389/fimmu.2023.1084531, PMID: 36911691 PMC9992203

[16] Liu X Ren B Fang Y Ren J Wang X Gu M . Comprehensive analysis of bulk and single-cell transcriptomic data reveals a novel signature associated with endoplasmic reticulum stress, lipid metabolism, and liver metastasis in pancreatic cancer. J Transl Med. (2024) 22:393. doi: 10.1186/s12967-024-05158-y, PMID: 38685045 PMC11057100

[17] Poirion OB Jing Z Chaudhary K Huang S Garmire LX . DeepProg: an ensemble of deep-learning and machine-learning models for prognosis prediction using multi-omics data. Genome Med. (2021) 13:112. doi: 10.1186/s13073-021-00930-x, PMID: 34261540 PMC8281595

[18] Yi M Zheng X Niu M Zhu S Ge H Wu K . Combination strategies with PD-1/PD-L1 blockade: current advances and future directions. Mol Cancer. (2022) 21:28. doi: 10.1186/s12943-021-01489-2, PMID: 35062949 PMC8780712

[19] Kabir AU Zeng C Subramanian M Wu J Kim M Krchma K . ZBTB46 coordinates angiogenesis and immunity to control tumor outcome. Nat Immunol. (2024) 25:1546–54. doi: 10.1038/s41590-024-01936-4, PMID: 39134750 PMC13355241

[20] Huangfu L Li R Huang Y Wang S . The IL-17 family in diseases: from bench to bedside. Signal Transduct Target Ther. (2023) 8:402. doi: 10.1038/s41392-023-01620-3, PMID: 37816755 PMC10564932

[21] Li Y Zhou T Liu Z Zhu X Wu Q Meng C . Air pollution and prostate cancer: Unraveling the connection through network toxicology and machine learning. Ecotoxicol Environ Saf. (2025) 292:117966. doi: 10.1016/j.ecoenv.2025.117966, PMID: 40022828

[22] Wang Q Qiao W Zhang H Liu B Li J Zang C . Nomogram established on account of Lasso-Cox regression for predicting recurrence in patients with early-stage hepatocellular carcinoma. Front Immunol. (2022) 13:1019638. doi: 10.3389/fimmu.2022.1019638, PMID: 36505501 PMC9726717

[23] Li X Li X Qin J Lei L Guo H Zheng X . Machine learning-derived peripheral blood transcriptomic biomarkers for early lung cancer diagnosis: Unveiling tumor-immune interaction mechanisms. Biofactors. (2025) 51:e2129. doi: 10.1002/biof.2129, PMID: 39415336 PMC11681315

[24] da Silva AR Gunawan F Boezio GLM Faure E Theron A Avierinos JF . egr3 is a mechanosensitive transcription factor gene required for cardiac valve morphogenesis. Sci Adv. (2024) 10:eadl0633. doi: 10.1126/sciadv.adl0633, PMID: 38748804 PMC11095463

[25] Zhang Q Cheng S Wang Y Wang M Lu Y Wen Z . Interrogation of the microenvironmental landscape in spinal ependymomas reveals dual functions of tumor-associated macrophages. Nat Commun. (2021) 12:6867. doi: 10.1038/s41467-021-27018-9, PMID: 34824203 PMC8617028

[26] Kwon Y Kim M Kim Y Jeong MS Jung HS Jeoung D . EGR3-HDAC6-IL-27 axis mediates allergic inflammation and is necessary for tumorigenic potential of cancer cells enhanced by allergic inflammation-promoted cellular interactions. Front Immunol. (2021) 12:680441. doi: 10.3389/fimmu.2021.680441, PMID: 34234781 PMC8257050

[27] Hong W Zhang Y Wang S Li Z Zheng D Hsu S . RECQL4 Inhibits Radiation-Induced Tumor Immune Awakening via Suppressing the cGAS-STING Pathway in Hepatocellular Carcinoma. Adv Sci (Weinh). (2024) 11:e2308009. doi: 10.1002/advs.202308009, PMID: 38381090 PMC11040365

[28] Yang Y Zheng W Tan W Wu X Dai Z Li Z . Injectable MMP1-sensitive microspheres with spatiotemporally controlled exosome release promote neovascularized bone healing. Acta Biomater. (2023) 157:321–36. doi: 10.1016/j.actbio.2022.11.065, PMID: 36481504

