# Supplementary figures and images for: EGR3 as a dual tumor-immune regulator: a machine learning-driven prognostic target for cold breast cancer
Source: Front Immunol. 2025 Dec 15;16:1627133. doi: 10.3389/fimmu.2025.1627133 (PMC12745386; doi:10.3389/fimmu.2025.1627133)

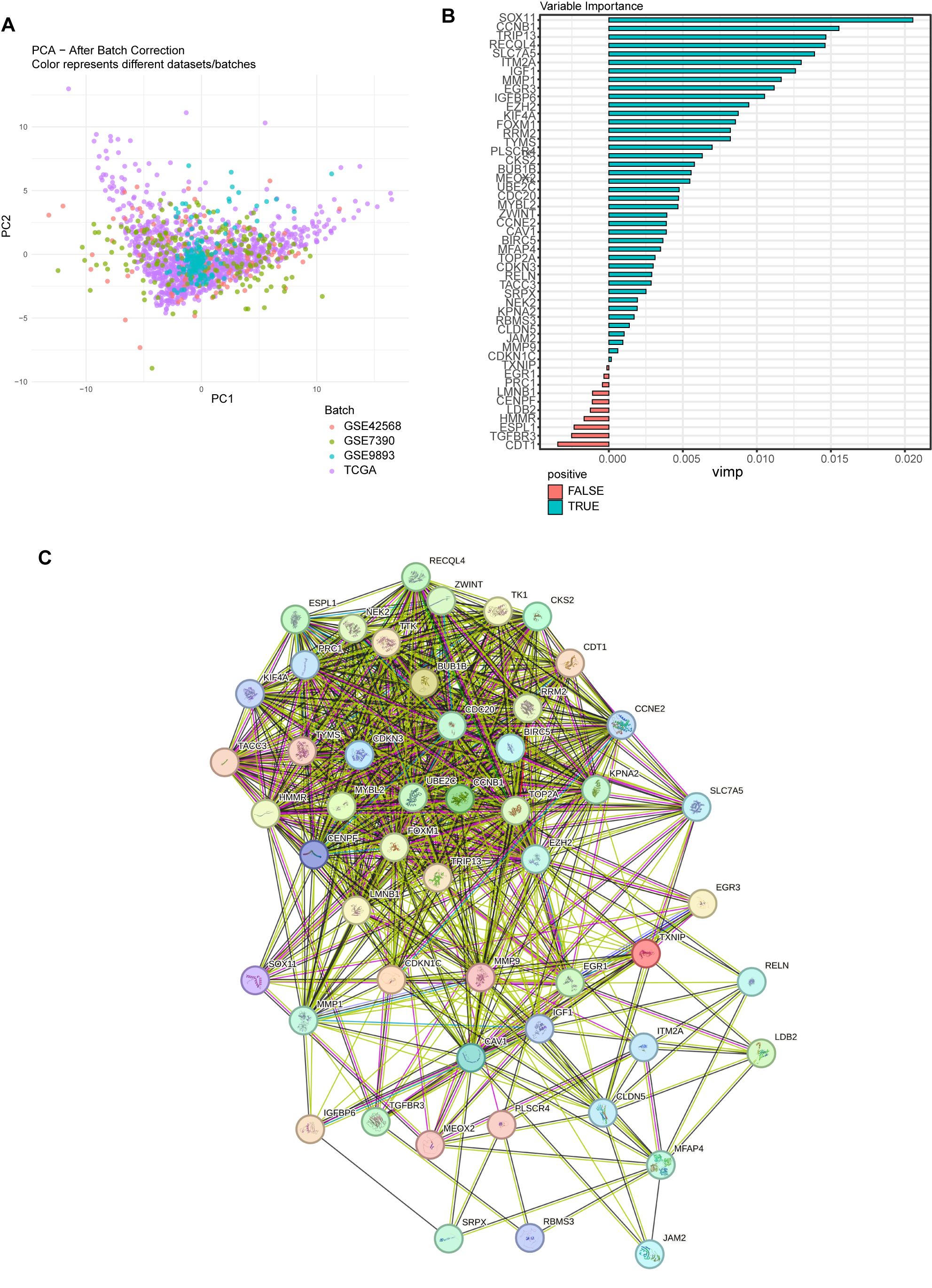

Supplement: Supplementary Figure 1 — Display of prognostic-associated genes. (A) Visualization of machine learning-relevant genes by VIMP measure. (B) STING protein-protein interaction (PPI) network visualization of machine learning-identified genes. [file Image1.tif]

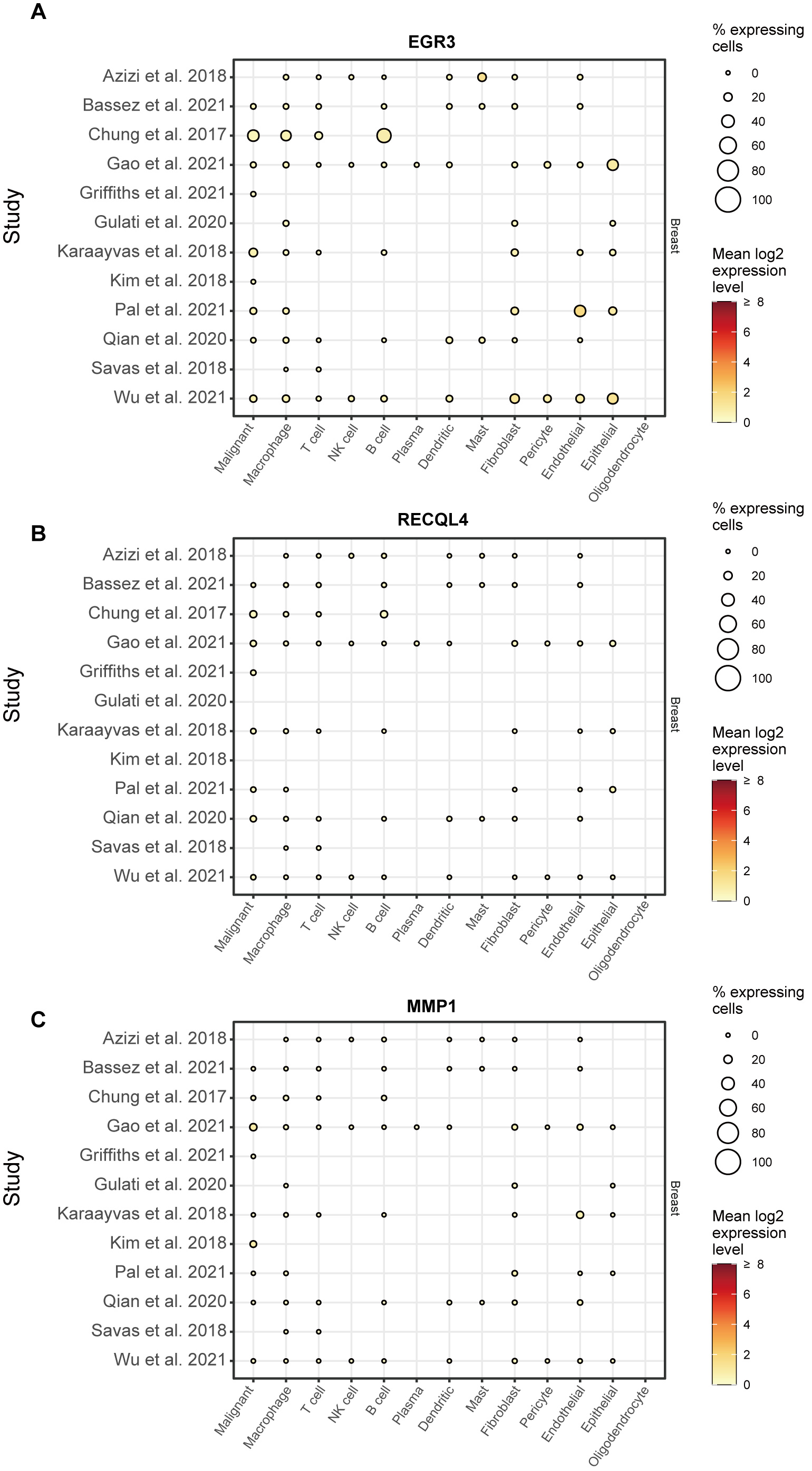

Supplement: Supplementary Figure 2 — Single-cell transcriptional landscape of prognostic signature genes. (A–C) Multi-dataset single-cell profiling of EGR3, RECQL4, and MMP1 expression across tumor microenvironment cell types. [file Image2.tif]

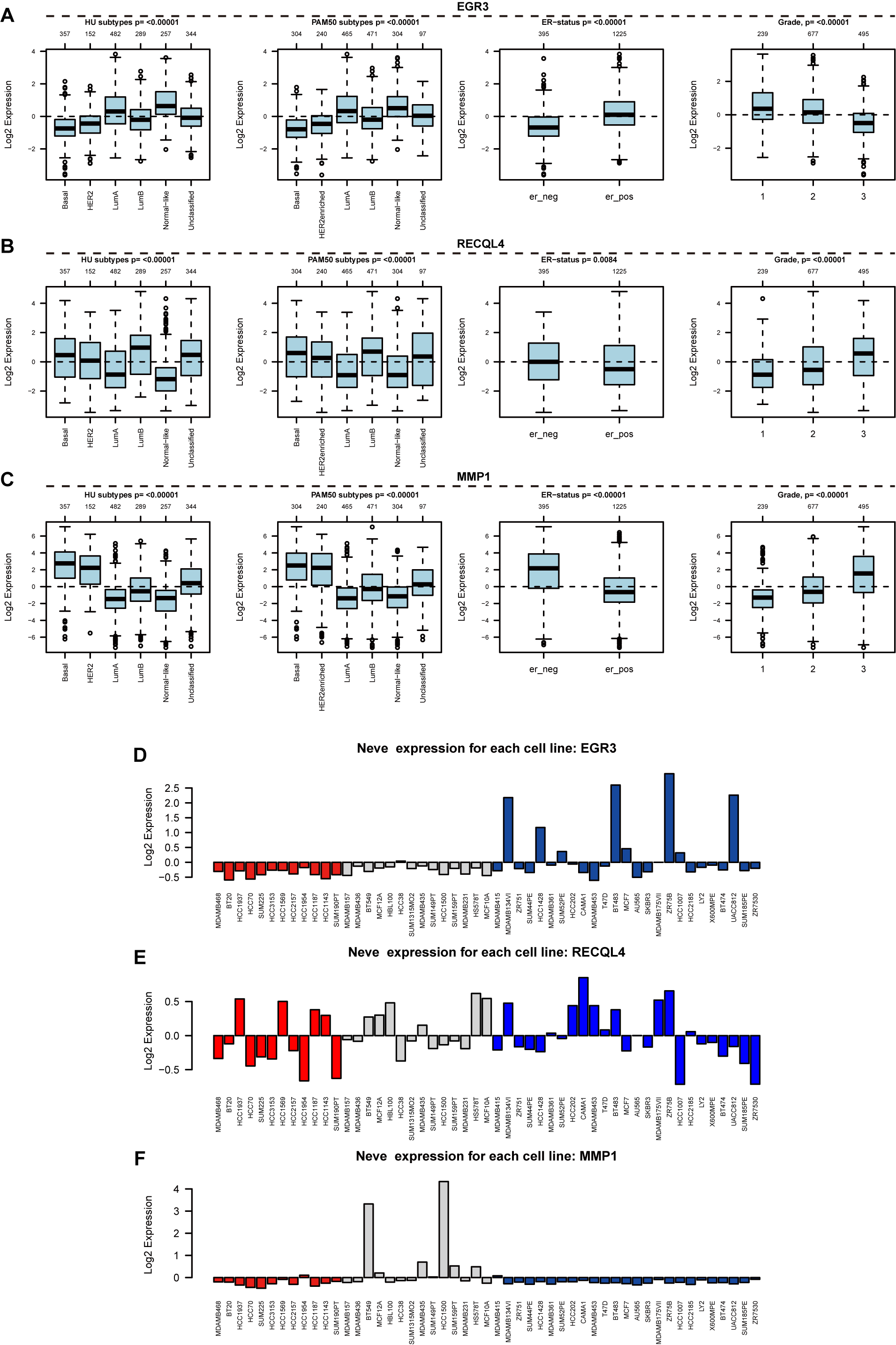

Supplement: Supplementary Figure 3 — Prognostic signature genes across different breast cancer subtypes: clinical implications. (A–C) Distinct expression patterns of EGR3, RECQL4, and MMP1 across HU classification, PAM50 subtypes, ER status, and histological grades. (D–F) Differential expression profiles of EGR3, RECQL4, and MMP1 across breast cancer cell lines. [file Image3.tif]

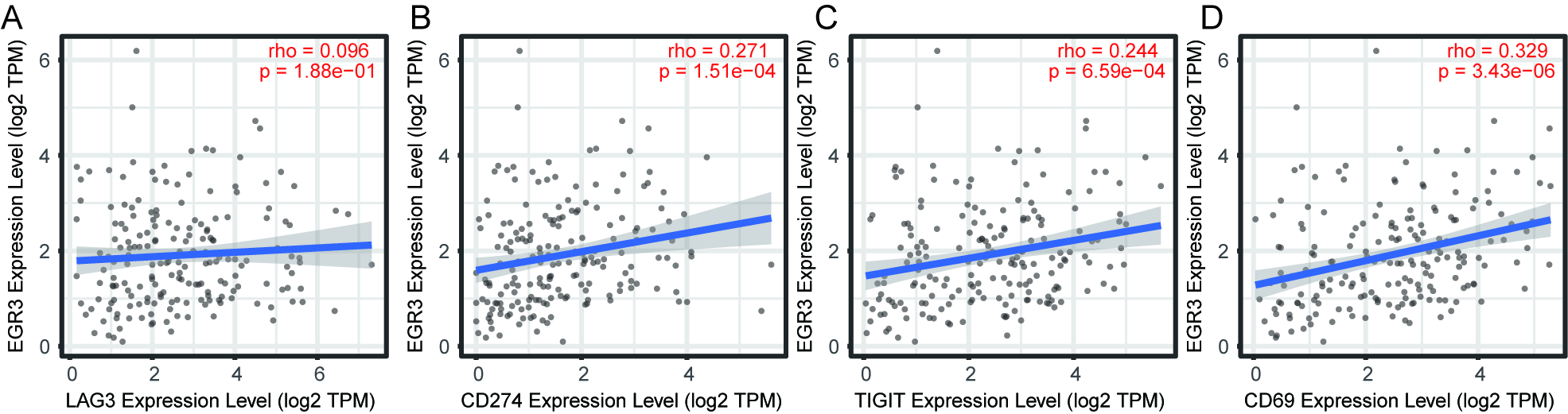

Supplement: Supplementary Figure 4 — Correlation of EGR3 with immune-related molecules. (A) Correlation between EGR3 and LAG3. (B) Correlation between EGR3 and CD274. (C) Correlation between EGR3 and TIGIT. (D) Correlation between EGR3 and CD69. [file Image4.tif]
